# The Prevalence of Reproductive Tract Infections and Sexually Transmitted Diseases Among Married Women in the Reproductive Age Group in a Rural Area

**DOI:** 10.4103/0970-0218.45376

**Published:** 2009-01

**Authors:** Savita Sharma, BP Gupta

**Affiliations:** 1 Assistant Professor, Department of Community Medicine, Indira Gandhi Medical College, Shimla, Himachal Pradesh, India; 2 Former Professor and Head, Department of Community Medicine, Indira Gandhi Medical College, Shimla, Himachal Pradesh, India

## Introduction

Reproductive tract infections (RTIs) and sexually transmitted diseases (STDs) represent a major public health problem in developing countries.(1) The consequences of RTIs are numerous and potentially devastating. These include postabortal and puerperal-sepsis, ectopic pregnancy, fetal and perinatal death, cervical cancer, infertility, chronic physical pain, emotional distress, and social rejection of women.(2) In Indian community-based studies, the range of self-reported morbidity has been reported to vary from 39–84%.(3,4) Most of the Indian studies in the field of reproductive health care are based on clinical examination and a few are based on laboratory tests.(5)

This community-based cross-sectional study of the prevalence of RTIs gives some indication of the likely burden of the disease in the population studied and highlights some associated factors.

## Methodology

This study was conducted with the objective of assessing the prevalence of various RTIs among married women in the reproductive age group of 15-44 years old in a rural area of the district of Sirmour (H.P.) during December 2001–March 2002. The sample size was calculated by taking the prevalence of RTIs as 50% and the permissible level of error as 10% and was computed using 400 women in the reproductive age group of 15–44 years old. By simple random technique, three sub-centers falling under PHC Sarahan in the Pachhad block of the district of Sirmour were chosen. All the women who had ever been married in the age group of 15–44 years old were interviewed through domiciliary visits in all the villages and hamlets falling under the three sub-centers in this hilly and rugged terrain. For cultural and social reasons, unmarried women and girls were not included; all the pregnant and puerperal women were also excluded. A pre-tested and semi-structured proforma was used during the household surveys. The syndromes related to women such as vaginal discharge, genital ulcer disease, lower abdominal pain, and inguinal bubo based on the syndromic approach as recommended by the Government of India, Ministry of Health and Family Welfare for the management of RTIs/STDs were considered. The case definitions of these syndromes as recommended by National AIDS Control Organization were strictly followed for diagnosis and treatment of patients. Each woman was interviewed in private about her socio-demographic and reproductive history, current and past symptoms affecting the reproductive tract, and past sexual behaviour, etc. All women suffering from RTIs were given prescription slips in accordance with syndromic management flow charts and were asked to collect free treatment from PHC, Sarahan.

## Results

A total of 452 women were interviewed and 235 of them were found to be suffering from RTIs giving a prevalence of 51.9%. The trend in relation to age showed maximum prevalence in the 25–34 year old age group (63.6%). The difference was statistically significant (X^2^=17.46, *P* <.001) [Table 1].

**Table 1:** Socio-demographic characteristics of the sexually active married women in the reproductive age group of 15 to 44 years

	Number of participants	Number of symptomatic patients	%	Level of significance
Age group				
<25	208	89	42.8	-
25–34	184	117	63.6	x^2^=17.46
>=35	60	29	48.3	*P* < .001
Marital status				
Unmarried	0	0	0	-
Married	445	233	52.4	-
Divorced/widowed	7	2	28.6	-
Education				
Illiterate	72	52	72.2	-
Primary	169	97	57.4	x^2^=31.11
Middle	152	70	46.1	*P* < .001
High and secondary	52	15	28.9	-
Graduate and above	7	1	14.3	-
Occupation				
Housewife	152	38	25.0	-
Agriculture	278	192	69.1	x^2^=39.85
Service	15	2	13.3	*P* < .001
Others	7	3	42.9	-
Number of children				
None	52	12	23.1	-
1–2	7	152	59.1	x^2^=22.76
3	91	46	50.6	*P* < .001
4	36	18	50.0	-
5+	16	7	43.8	-
Contraception				
None	205	107	52.2	-
Condom	30	4	13.3	-
OCP	12	2	16.7	x^2^=40.17
IUD	160	99	61.9	*P* < .001
Tubectomy	45	23	51.1	-
DMPA	0	0	0	-

DMPA = Depot -medroxyprogestrone acetate; IUD = Intrauterine device; OCP = Oral contraceptive pills

Divorcees and widows (n = 7) who were in small number did not report any non-marital sexual relationships and the prevalence of RTIs was less (28.6%) as compared with married women (52.4%). Unmarried women and girls who were not included in the study due to social constraints could introduce some bias.

The prevalence of RTIs was found to be higher in illiterate women (72.2%) and it showed a decreasing trend with an increase in the level of education. Of the 278 women (61.5%) who were working in the fields and rearing domestic animals, 192 (61.7%) of them had one or more RTIs. Housewives who constituted 152 (33.6%) of the study subjects had less prevalence of RTIs (25%).

The prevalence of RTIs was far less in women who had no children (23%) as compared with women who had children (*P* < .001). A higher prevalence (59.1%) was found among women who had one or two children and did not vary much during subsequent deliveries (50.6%, 50%, 43.8%).

More than 50% of the women studied were not using any contraceptives at the time of the survey and 107 women (52.2%) had an RTI. A total of 61.9% of the women using an intrauterine device and the 51.1% of women who had tubectomies had RTIs. The prevalence of RTIs among women using oral contraceptive pills and condoms was less (13.3%, 16.7%).

The most common presentation among women was vaginal discharge (51.9%) followed by lower abdominal pain. Less than 2% of women had genital ulcer disease and inguinal lymphadenopathy. All the women complaining of lower abdominal pain also had a history of vaginal discharge [Table 2].

**Table 2:** The prevalence of symptoms among the symptomatic patients and in the study population

	Symptomatic patients n = 235 (%)	Study population n = 452 (%)
Vaginal discharge	235 (100.0)	51.9
Lower abdominal pain	90 (38.3)	19.9
Genital ulcer disease	5 (2.1)	1.1
Inguinal lymphadenopathy	2 (0.8)	0.4

Apart from vaginal discharge, which was the most common symptom (51.9%), many women were also suffering from other reproductive morbidities like menstrual problems, lower backache, and burning micturition. Some women reported having more than one reproductive morbidity.

## Discussion

From this study, the prevalence of RTIs was 51.9%, which is high probably due to a lack of awareness and the inaccessibility of health care services in this hilly and far flung area. Others have reported similar high rates of RTIs in community-based studies in developing countries.(3,5–7) In a study among rural Indian women,(3) a high prevalence of bacterial vaginosis (62%), candidiasis (34%), trichomoniasis (13.98%), and syphilis (10.5%) was observed. In Papua New Guinea,(5)59% of the women studied had lab-confirmed STDs in a community-based survey. The prevalence of RTIs/STDs was found to be 49%(6) in a rural area of the district of Agra (U.P.) while 70% of the women studied in a rural area of Haryana were found to be suffering from RTIs.(7) Comparatively, low prevalence was observed by studies conducted in slum and rural areas of Chandigarh (21.6%, 17.7%).(8,9)

In this study, maximum prevalence was found in the age group of 25–34 years old - a period of maximum sexual and reproductive activity. A similar observation was made in this age group in a study conducted in Agra.(6) A study on community-level health-education intervention(10) reported an improvement in the level of awareness among both men and women regarding RTIs and STDs and also revealed an eight-fold increase in their clinic attendance. In this study, the high case load has been found based on the syndromic approach and steps should be taken for appropriate management of these cases in view of the high potential for the spread of HIV/AIDS. There is also a need to conduct further studies to assess various behavioural and socio-demographic factors, predisposing these women to the risk of RTIs and STDs.
